# Synchrotron radiation transmission by two coupled flat microchannel plates: new opportunities to control the focal spot characteristics

**DOI:** 10.1107/S1600577521012893

**Published:** 2022-01-19

**Authors:** M. I. Mazuritskiy, A. M. Lerer, A. Marcelli, S. B. Dabagov

**Affiliations:** a Physics Department, Southern Federal University, Sorge Str. 5, 344090 Rostov-on-Don, Russian Federation; b INFN – Laboratori Nazionali di Frascati, Via Enrico Fermi 54, 00044 Frascati, Italy; c RICMASS, Rome International Center for Materials Science Superstripes, 00185 Rome, Italy; d Istituto Struttura della Materia, CNR, Via del Fosso del Cavaliere 100, 00133 Rome, Italy; e RAS P. N. Lebedev Physical Institute, Leninsky Pr. 53, 119991 Moscow, Russian Federation; f NR Nuclear University MEPhI, Kashirskoe Sh. 31, 115409 Moscow, Russian Federation

**Keywords:** X-ray focusing, microchannel plate, X-ray waveguides, X-ray optics, X-ray diffraction

## Abstract

An improved theoretical model to calculate the focal spot properties of coherent synchrotron radiation (SR) soft X-ray beams by combining and aligning two microchannel plates (MCPs) is presented. The diffraction patterns of the radiation behind the MCP system are simulated in the framework of the electrodynamical model of the radiation emission from two-dimensional finite antenna arrays. Simulations show that this particular optical device focuses the soft X-ray radiation in a circular central spot with a radius of ∼4 µm. The study points out that such MCP-based devices may achieve micrometre and sub-micrometre spot sizes as required by many applications in the soft X-ray range. Finally, based on experimental and theoretical results of the radiation transmission by this MCP-based device, a new method to characterize the spatial properties of brilliant SR sources is discussed.

## Abstract 2

Transmission properties and diffraction patterns generated by synchrotron radiation at the exit of an assembled couple of microchannel plates (MCPs) are inivestigated. A theoretical model to simulate the patterns and properties of the soft X-ray beam emerging from this couple of MCPs is presented and discussed.

## 1. Introduction

Third-generation synchrotron radiation facilities and free-electron lasers (FELs) are brilliant sources of coherent X-rays open to a interdisciplinary scientific community that allow many new experiments to be performed. In addition to undulator devices installed at third-generation synchrotron radiation (SR) facilities, FELs are unique radiation sources that deliver narrow-band coherent radiation beams. Operational SR facilities are characterized by wide spectral ranges delivering radiation from the IR/THz range up to the ‘nm domain’ (Tsuji *et al.*, 2004 ▸).

In addition to the brilliance, an important property of the X-ray beam is the coherence, which characterizes the correlation between two electromagnetic waves at different points in space in both transverse and longitudinal directions. As known, at high spatial coherence the beam divergence tends to 2λ/*d*, where λ and *d* are the wavelength and diameter of the radiation beam, respectively (Hiroyuki, 2002 ▸).

The technologies to obtain a micrometre-size soft X-ray spot are described by Tsuji *et al.* (2004 ▸). There, a comparison of various combinations of slits and collimators with polycapillary-based optical systems as grazing-incidence optics is discussed. The main characteristics of polycapillary spectroscopic devices are size and divergence of the transmitted beam. These quite compact diffractive optics are applied in X-ray spectroscopy and in many other analytical techniques obtaining small spots (from several micrometres to tens of nanometres) or a high photon density at the focal point or both, *i.e.* in the micrometre and sub-micrometre ranges (Pfeiffer *et al.*, 2002 ▸; Dabagov, 2003*a* ▸; Bukreeva *et al.*, 2006 ▸, 2010 ▸; Sun *et al.*, 2009 ▸; MacDonald, 2010 ▸; Dabagov & Gladkikh, 2019 ▸).

X-ray beams shaped by various polycapillary devices have been characterized since the 1990s (Kumakhov & Komarov, 1990 ▸; Bilderback *et al.*, 1994 ▸; Dabagov *et al.*, 1995*a* ▸; Dabagov, 2003*b* ▸; MacDonald & Gibson, 2000 ▸). Among polycapillary-based optical systems, microchannel plates (MCPs) are different compact devices allowing beam focusing and shaping. Studies of MCPs date back to the 1990s (Chapman *et al.*, 1990 ▸, 1993 ▸; Kaaret *et al.*, 1992 ▸; Nussey, 2005 ▸). The radiation propagation through MCPs is typically described within classical (ray) optics as multiple reflections from their channel’s internal walls. This approach, however, neglects the wave nature of the radiation and, in particular, the phase evolution of the propagating wave. This approach also cannot explain interference phenomena occurring while crossing different components (Dabagov *et al.*, 1995*b* ▸, 2000 ▸). Indeed, surface and bulk channeling of radiation in micro- and nano-guides in capillary-based systems have been described and observed (Dabagov & Uberall, 2007 ▸, 2008 ▸). In this work we analyze the radiation propagation in thin MCPs with narrow channels taking into account the phase evolution of the transmitted waves. The approach allows interference and focusing of X-ray beams behind different multichannel MCP configurations.

## 2. MCP optical systems

We have characterized several different MCPs, made by Si–Pb glass with composition (PbO)_0.7_(SiO_2_)_0.3_ manufactured by the Vladikavkaz Technological Center ‘Baspik’ (http://www.baspik.com/eng/products/nauka/) (Mazuritskiy *et al.*, 2019*b* ▸). We used relatively thin MCPs, *e.g.* 0.3–1.5 mm thick, with 10^4^–10^7^ hollow hexagonal-structured microchannels of equal length, extending through the whole thickness. The length-to-diameter ratio of a typical hollow microchannel spans from 40 to 150. Exhibiting rather high transmission efficiency (up to ∼60%) they are efficient waveguide optical systems. Moreover, MCP optical properties allow increasing the radiation density as well as shaping the beam (Mazuritskiy *et al.*, 2016*a* ▸,*b* ▸, 2017*a* ▸,*b* ▸, 2018 ▸, 2019*a* ▸). Although many experimental and theoretical investigations have been published on single flat or bent MCPs, no data have been published on assembled and aligned MCPs.

**Figure 1:**
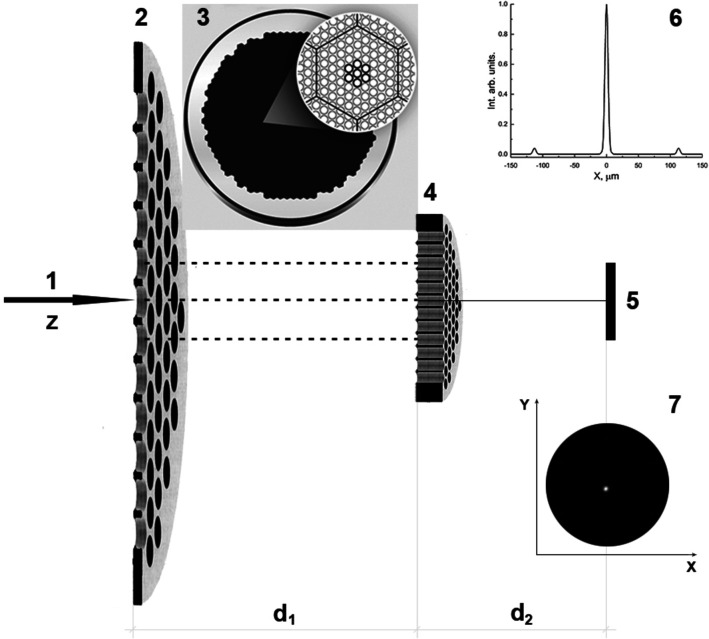
Scheme of two flat parallel coaxial MCPs for focusing of the X-ray beam; *d*_1_ is the distance between the two MCPs and *d*_2_ the distance between the detector and second MCP. 1 – primary radiation; 2 – first MCP; 3 (inset) – image of the capillary structure of both MCPs; 4 – second MCP; 5 – 2D detector of radiation; 6 (insert) – focal spot profile; 7 (insert) – focal spot cross-section.

In this work we discuss an original layout named Microchannel Aligned Assembled System (MAAS), which is formed by two different flat MCPs assembled such that their axis of the channels are maintained parallel. Fig. 1 ▸ shows the layout with the parallel coherent beam (1) propagating along the *z*-axis and two coaxial plates at the distance *d*_1_. In this layout, the primary beam – a monochromatic coherent plane wave with a Gaussian amplitude distribution in the transverse cross-section – is focused by the hollow microchannels of both MAAS MCPs. The distance *d*_1_ between two MCPs and the detector position at the distance *d*_2_ from the second MCP are the main parameters of the simulation. The *d*_2_ distance also determines the 2D-detector offset position (5).

## 3. The theoretical model

In the simulations of the MAAS device (Fig. 1 ▸), microchannel diameters as well as pitch sizes and periods may change to set the optimal parameters for focusing, interference, diffraction, *etc*. The model considers two MCPs of ideal hexagonal symmetry in the transverse cross-sections and with the symmetry axis along the *z*-axis. In the simulations, each MCP is characterized by its dispersion equation, optimized by suitable microchannels. The first MCP is used to shape the primary parallel beam (coherent or partially coherent). Behind the first MCP the beam becomes conical in shape, and this ‘secondary beam’ defines the radiation footprint on the surface of the second MCP, chosen thicker and with smaller microchannels.

Focusing depends on the energy, the refractive index of the MCP medium, the channel diameter, the number of illuminated microchannels, and the thickness. By changing the distance between the two MCPs, the parameters of the antenna array of the second MCP change the diffraction pattern. The radiation reaches the detector plane forming a pattern with an intense narrow center peak (‘zero position’) and weaker side peaks. Let us assume that the original radiation is represented by a monochromatic plane wave with a Gaussian intensity distribution in the transverse cross-section,

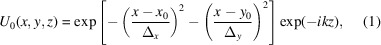

where *x*_0_, *y*_0_ are the coordinates of the beam center; Δ_*x*_, Δ_*y*_ are the parameters of the Gaussian function and *k* is the wavevector. We consider now the radiation propagating along the *z*-axis (Fig. 1 ▸) normal to the surfaces of two flat MCPs of the MAAS device. The diffraction field formed behind the first MCP is the radiation source (‘a secondary source’) of the second MCP. Moreover,

(i) Both devices have a hexagonal packing of hollow cylindrical microchannels characterized by microchannel radii and pitch sizes: *R*_1_ and *D*_1_ and *R*_2_ and *D*_2_.

(ii) The coaxial MCPs are set at the distance *d*_1_, while *d*_2_ (Fig. 1 ▸) is the distance between the second MCP and the detector.

(iii) Considering the hexagonal symmetry, the coordinates of the microchannel centers for both MCPs can be calculated using the following expression,

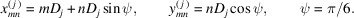

The microchannels are defined by ‘*m*,*n*’ in the *xy*-plate and *j* = 1 or 2 for the first and second MCP, respectively.

We evaluate the diffraction at the far zone in the frame of the Kirchhoff–Huygens approximation. The model rules out any interaction among MCP microchannels, and the wavefield emitted by each cylindrical channel of the *j*th MCP can be described as

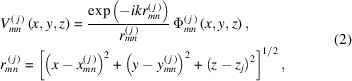

defined by the space distribution 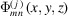 of the radiation pattern at the exit of each channel (*m*,*n*) of the *j*th MCP. The number of illuminated channels of the first MCP equals (2*M*_1_ + 1) × (2*N*_1_ + 1), while of the second equals (2*M*_2_ + 1) × (2*N*_2_ + 1). *M*_1_ and *N*_1_ are determined by the profile of the primary beam and the footprint on the front surface of the first MCP. At variance, due to the conical shape of the beam emerging from the first MCP (see Fig. 1 ▸), the values *M*_2_, *N*_2_ depend also on the distance *d*_1_. Within the MAAS framework, at a large *d*_1_ distance we have many illuminated channels on the front side of the second MCP. The footprint dimension, obviously, affects the interference at the second MCP exit. The electromagnetic field at this plane is due to the interference of the beams emerging by the channels of the first MCP,

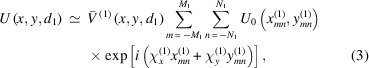




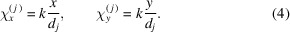

In our model we assume the radiation field constant inside each microchannel of the second MCP (multiple reflection mode),

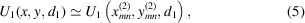

so that the final diffraction pattern at the detector plane can be calculated as follows,

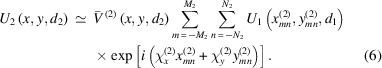

While all equations have been written for parallel flat MCPs with the central axes (*m* = *n* = 0) set along the *z*-axes, the model enables simulations for any ( 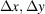 ) displacement of the first MCP as well as for any angular rotation respect to the initial position of ( 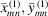 ),

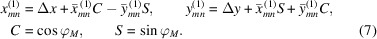

For the sake of simplicity the field at the exit of a single microchannel can be derived for a circular hole within an opaque screen. This model of propagation inside a circular hole well approximates a waveguide and the radiation field at the exit can be calculated using the following equation (Mazuritskiy & Lerer, 2015 ▸, 2016*b* ▸),

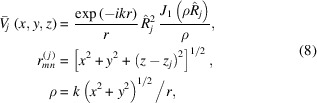

where 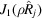 is the Bessel function and 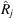 , the model parameter, is the effective radius of the microchannel hole. For a single MCP the value 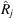 can be evaluated by comparing experimental and simulated diffraction patterns. The decrease of the MCP thickness, *i.e.* the channel length, can be described by a smaller radius 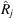 because this parameter defines the solid angle of the radiation spatial distribution at the microcapillary exit. Let us now examine the pattern of the radiation emerged by a single microchannel that can be applied also to any selected channel or set of channels within these assumptions:

(i) The validity of the complex propagation constants for the waveguide modes inside a circular hollow dielectric guide inhomogeneous in depth (see Mazuritskiy *et al.*, 2014 ▸).

(ii) The validity of the amplitudes of the waveguide modes within the Kirchhoff–Huygens approach.

(iii) The validity of the mode amplitudes at the waveguide output using the complex propagation constants.

(iv) The far-field angular distributions of the radiation from each waveguide using the Kirchhoff–Huygens method.

The electric field of the plane wave inside the waveguide (capillary) is

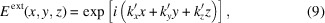

where 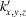 are the wavevector projections. The primary radiation field at the aperture of the waveguide and inside the waveguide can be written in a similar way,

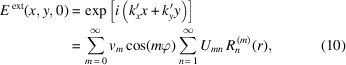

where

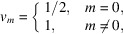

and 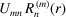 is the amplitude that depends on the radius of the wavefield distribution characterized by the indexes *m*,*n*. To calculate equation (10) we use the *M* and *N* maxima corresponding to the *m* and *n* indexes. *M* is usually not greater than 3, while *N* is the maximum number of the wave modes accepted by the capillary. Taking into account the orthogonal properties of the function we can rewrite equation (10) as

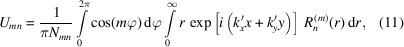




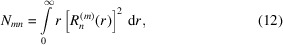

where 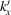 = 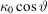 , 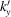 = 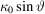 , *x* = 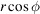 , *y* = 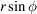 and κ_0_ is the perpendicular component of the radiation wavevector to the capillary axis at the entrance. In addition, we assume the wavefield to be confined inside the microcapillary. In this approximation, integrating equations (10)–(12) over the segment and assuming for the transverse wavenumber, we obtain the following expressions,

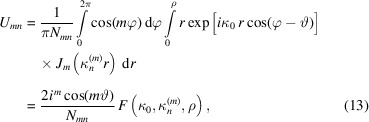




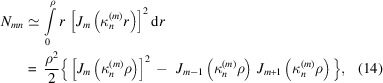




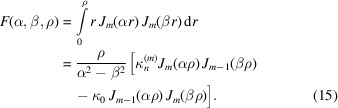

At the capillary exit the field is calculated as the sum of the waves,

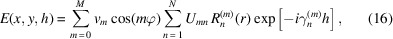

where 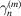 is the longitudinal wavenumber and *h* is the capillary length. The radiation field is defined as

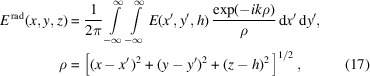

assuming

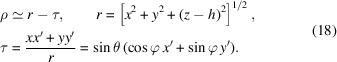

Using the spherical coordinates *r*, θ, φ, the radiation field can be rewritten as

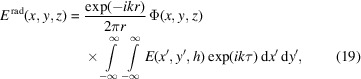




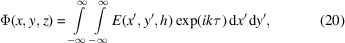

and finally, replacing the field in equations (19)–(20) with that of equation (16), we obtain

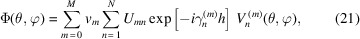

where

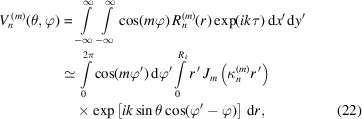

and this expression is equal to

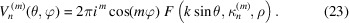




## 4. Results and discussion

Using the above model, simulations were performed for a primary parallel beam with a cross section of 100 µm × 100 µm at an energy of 94 eV. The parameters of the calculations correspond to the energy and the characteristics associated with the BESSY II (Berlin) and ELETTRA (Trieste) experimental parameters.

In the experiments, the primary beam hits the first MCP with hexagonal packing, cylindrical channels of 10 µm diameter and 12 µm pitch, and with a ratio thickness to microchannel diameter of 40. The second MCP was thicker (0.5 mm) with a smaller microchannel diameter (3.4 µm), 4.0 µm pitch, and ratio thickness to microchannel diameter of 150. The distance *d*_1_ between MCPs varied between 100 and 300 mm, while *d*_2_ was in the range 10–30 mm (see Fig. 1 ▸).

**Figure 2:**
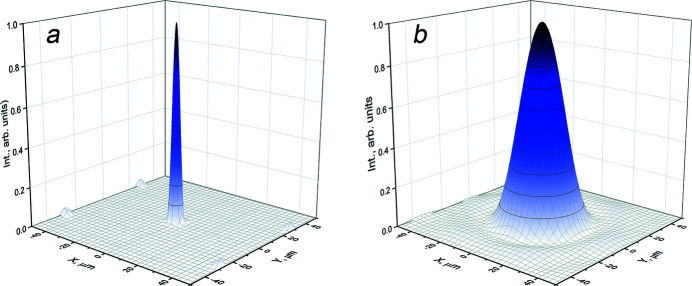
Spatial SR intensity distributions behind the device: (*a*) at the exit of the second MCP (just beyond the second plate); (*b*) at the entrance of the second MCP with 3.4 µm channel diameter and 4.0 µm pitch.

For these simulations the 2D-detector was placed perpendicularly to the direction of the primary beam (CiPo-Beamline; Elettra Synchrotron https://www.elettra.trieste.it/elettra-beamlines/cipo.html) and characterized by 10 µm spatial resolution (Marcelli *et al.*, 2018 ▸). Fig. 2 ▸ shows the simulated diffraction patterns for this layout. Panel (*a*) in Fig. 2 ▸ shows the radiation spatial distribution at the second MCP exit. The simulations of the radiation transmitted by the MAAS device show a minimum circular focal spot of ∼4 µm.

The diffraction pattern at the far-zone in Fig. 2 ▸(*a*) (at the exit of the second MCP) shows a highly intense and small divergent peak demonstrating that the SR beam transmitted by the two MCPs is concentrated at the center of the transverse space. In an ideal layout with two hexagonal MCPs perfectly aligned along the *z*-axis, this artificial photonic device works as a condenser of the primary parallel beam, but at the same time as an efficient diffractive X-ray optical device forming a constructive interference of the transmitted radiation.

Regarding its focusing efficiency, at 94 eV the field at the first MCP exit [Fig. 2 ▸(*b*)] exhibits a wide conical distribution. The secondary radiation source generated by this MCP changes propagating towards the second becoming the primary source at the entrance plane of the second MCP. The radiation change between MCPs redistributes the intensity and reshapes the beam. Actually, the primary parallel SR beam is different from the beam hitting the second MCP. The difference becomes more evident for a thicker MCP, since longer microchannels reduce the angular and spatial distributions of the radiation at the exit of the second MCP.

**Figure 3:**
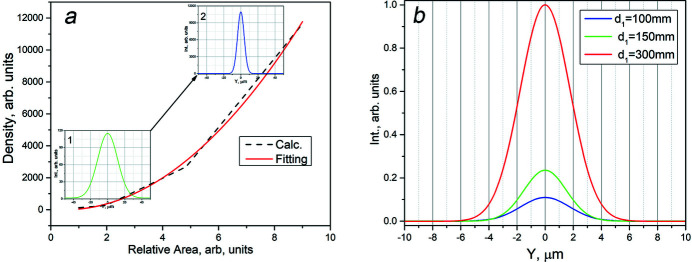
(*a*) The radiation density at the exit of the second MCP as a function of the primary beam cross-section (squared radius *R* ^2^). Inset 1 – for 100 µm; inset 2 – for 300 µm. (*b*) Comparison of beam profiles for different MCPs distances in the range 100–300 mm.

Simulations for a circular-shaped primary beam of 100–300 µm radius in cross section have revealed an optimal interpolated distance *d*_1_, at which the illuminated area of the second MCP is larger. For such a layout we maximize the number of microchannels transmitting radiation. Obviously, the MAAS device is more efficient in generating a brilliant radiation source either in a condenser or in a diffractive regime. Within the wave approach, this device exhibits a strong correlation of the diffraction pattern at the second MCP exit with the illuminated area of the first MCP. In Fig. 3 ▸(*a*), looking at the radiation intensity after the second MCP exit as a function of the primary radiation, the dashed line is the result of the calculations, while the red line is the fit.

The pattern central maximum can be fit by a 2D-Gaussian function, *i.e.* insets 1 and 2 for *X* = 1 (*R* = 100 µm) and *X* = 9 (*R* = 300 µm), respectively. The radiation densities on the *Y*-axis are calculated as the ratio of the radiation intensity to the Gaussian profile area. As shown in Fig. 3 ▸(*a*), the fit, which represents the radiation density behind the optical system versus the profile area of a primary beam, is well described by a second-order polynomial curve.

The pattern is the result of the interference of waves emitted by each pair of symmetrical microchannels of the first MCP having the same phases at the center of the second MCP. Thus, the first plate shapes the primary beam, *i.e.* the radiation hitting the surface of the second MCP. For a defined layout and symmetry properties the observed interference pattern also depends by the spatial coherence of the incoming beam.

Devices working as diffractive optical elements allow controlling the radiation density, redistributing the radiation in a different way compared with the condensing regime. Actually, the presence of two MCPs makes possible the redistribution of the condensed radiation maximum at the focal position because of the interference of the radiation. A more concentrated and narrower central maximum surrounded by high-order contributions occurs, the latter reflecting the symmetry of the device, that in our case is hexagonal. Considering a coherent primary beam, this MCP system should be optimized to obtain an interference pattern at the focal plane with a well defined intense central peak.

Fig. 3 ▸(*b*) shows the profiles of the central peak for different distances between the MCPs. We changed the illuminated area at the entrance surface of the second MCP changing the distance *d*_1_ (see Fig. 1 ▸). At large distances, *i.e.* at high *d*_1_ value, the illuminated area on the second MCP contains more microchannels [Fig. 3 ▸(*b*)]. The image shows the profiles of the intensity distribution of the radiation at the exit of the second MCP for different distances *d*_1_ in the range 100–300 mm. The flux increases of about one order of magnitude, while the FWHM of the Gaussian distributions remains constant as expected for a diffractive optics [Fig. 3 ▸(*b*)].

In our case, the interference of waves has a maximum at large distance, *i.e.* the radiation intensity at the center exhibits also a density gain. In these simulations we assumed a fully coherent beam (*i.e.* 100% degree of coherence) becoming partially coherent while propagating along the channels of the MCPs. With our model we could simulate various beam conditions including the coherence fraction.

The model allows also to simulate patterns at resonance detuning, considering small shifts of the MCP in the transversal plane (*xy*) and small angular rotations of the second MCP around the *z*-axis of the system. At any non-symmetrical MCPs-position the model shows a reduced contrast of the diffraction pattern as well as a broadening of the main peak. The latter exhibits the maximum density of the radiation on the *z*-axis only for a full axial symmetry of the field because of the constructive interference of waves in the far-zone after the second MCP.

Simulations for ideal layouts showed a clear reduction of the intensity (roughly a factor of 2–3) along the *z*-axis for a linear translation of MCPs of the order of tenths of a micrometre in the transversal plane (*xy*). We may hypothesize a decrease of the radiation density of the same order, which should take place for an (ideal) angular rotation of 2–3 mrad of the MCPs out of the symmetry position around the *z*-axis. However, in a ‘real’ device a full decoupling of rotations and translations is never straightforward, and any ‘action’ may result in much larger effects in terms of contrast and broadening of the experimental features. Moreover, in real MAAS devices the distance between the two MCPs cannot be changed, and a precise alignment depends also on this parameter. At smaller distances between the two MCPs, due to their hexagonal symmetry, the device could be sensitive even to ‘small’ rotations within the 60° rotation range.

In the energy range investigated, the transmission efficiency of this focusing device is about 32%. This evaluation is based on experimental data collected using a single flat MCP at the same energy that exhibits an efficiency of 57% (Mazuritskiy *et al.*, 2019*a* ▸). The results of this model point out that a device based on a pair of MCPs may be highly efficient in the soft X-ray range because of the low intensity loss caused by radiation absorption of the microchannel walls when precise alignment conditions for both MPCs are fulfilled.

**Figure 4:**
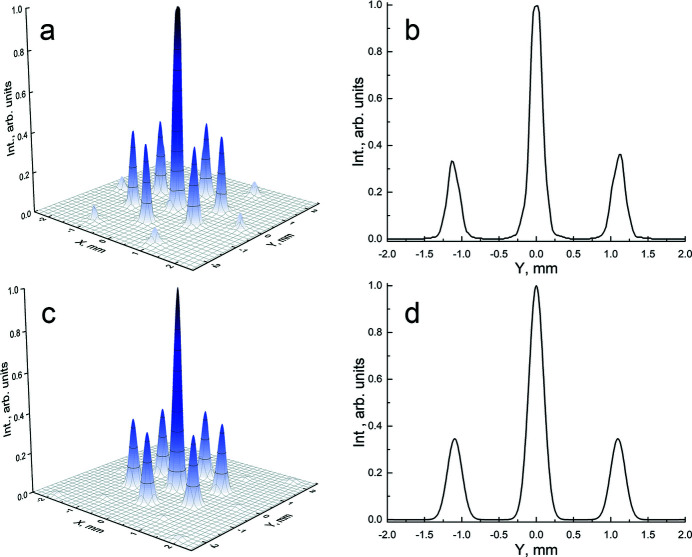
Single MCP diffraction pattern and beam profiles in the selected plane at 94 eV. (*a*, *b*) Experimental data; (*c*, *d*) simulated distributions.

To test the theoretical model and to compare these results with previous experiments performed using a single MCP, we also carried out simulations of X-ray diffraction at 94 eV. Simulations of an MCP, 0.3 mm thick with microchannels of diameter 3.4 µm and 4.2 µm pitch, are compared with experimental data in Fig. 4 ▸. These data at high spatial resolution were collected at the Reflectometer UHV station of the SR facility of BESSY II (Sokolov *et al.*, 2014 ▸, 2018 ▸). In this experiment the detector was set in the perpendicular plane at 310 mm distance from the MCP, whose front surface was set perpendicular to the primary SR beam. In this layout, the pattern of the diffracted radiation is characterized by an intense central peak, a set of first-order contributions and weak contributions of the second diffraction order. The comparison confirms the reliability of the model to describe the behavior of MAAS devices.

In our device the field at the exit of the first MCP has a hexagonal symmetry. Due to this geometry for defined parameters and dimensions of the MCPs, estimations provide the validity of the plane wave approximation behind the first MCP that propagates toward the second MCP. Considering a distance of several centimetres (*e.g.* from 10 to 30 cm) the divergent radiation exiting from one channel of the first MCP interferes with the radiation emerging from the adjacent channels. If we consider an entrance cross-section of 100 µm × 100 µm for which many channels are illuminated, the structured radiation field created by the first MCP illuminates the front surface of the second MCP. The structured pattern at the exit is quasi-homogeneous at the entrance of the second MCP, *i.e.* each channel produces a beam that at a large distance (*e.g.* from 10 to 30 cm) illuminates hundreds of channels of the second MCP.

At variance, for a device with much smaller distances between plates (*e.g.* a few mm), the structured illumination produced by the first MCP may generate different characteristics patterns.

## 5. Conclusion

This work presents the behavior of a new optical layout named Microchannel Aligned Assembled System (MAAS) based on a couple of two flat MCPs simulating the transmission of synchrotron radiation inside this device. We introduced a theoretical model built from a two-dimensional finite antenna array consisting of non-interacting emitters, which in our case are the MCP cylindrical microchannels acting as waveguides. The diffraction patterns of the primary synchrotron radiation transmitted through this device were calculated. In the soft X-ray range this waveguide model reproduces the focusing of the radiation from this assembled couple of MCPs. The device we characterized combines two different flat MCPs: the first with microchannels with a larger diameter that guarantees a high transmission while shaping the beam; and the second with narrower and longer microchannels that focuses the radiation at the detector plane. In this way it may behave as an optical condenser or a diffractive optics.

The analysis of the radiation distribution at its exit highlights the possibility to control macroscopic properties of the parallel or quasi-parallel primary beam. Moreover, using the theoretical model we introduced, the analysis of patterns generated by properly aligned couples of MCPs could be used to evaluate the coherent fraction of a SR source or a FEL.
